# Relationship Between Filler Type, Thermomechanical Properties, and Aging of RTV Silicone Foams

**DOI:** 10.3390/polym17141998

**Published:** 2025-07-21

**Authors:** Xavier M. Torres, John R. Stockdale, Adam Pacheco, Shelbie A. Legett, Lindsey B. Bezek, Bart Benedikt, Andrea Labouriau, Santosh Adhikari

**Affiliations:** 1 C-CDE: Chemical Diagnostics and Engineering Group, Los Alamos National Laboratory, Los Alamos, NM 87545, USA; xtorres@lanl.gov (X.M.T.); jstockdale@lanl.gov (J.R.S.); apacheco@lanl.gov (A.P.); slegett@lanl.gov (S.A.L.); lbezek@lanl.gov (L.B.B.); andrea@lanl.gov (A.L.); 2 W-13: Advanced Engineering Analysis, Los Alamos National Laboratory, Los Alamos, NM 87545, USA; bart_bebedikt@lanl.gov

**Keywords:** room temperature vulcanizing foams, diatomaceous earth, fumed silica, PDMS, carbon nanofibers, tin catalyst

## Abstract

Room-temperature vulcanizing (RTV) silicone foams are used in many industrial applications that require the material to perform over long time periods. However, mechanical properties tend to deteriorate when these foams age under a compressive load. The chemical aging is attributed to the presence of unreacted functional groups of the prepolymers, residues from acid, and catalytically active tin (II) species. Here, an optimized thermal treatment of an RTV foam that achieves completion of curing reactions and deactivation of reactive species is proposed. Foams that were thermally aged for three months under compressive load showed no signs of compression set, indicative of the effectiveness of the implemented post-curing approach. In addition, the effects of fillers (diatomaceous earth, fumed silica, and carbon nanofibers) on thermomechanical properties were investigated. Tensile strength, tear strength, and thermal conductivity increased when these fillers were added to the unfilled RTV formulation, with carbon nanofibers (CNFs) being the most effective filler. Rheological studies of RTV formulations indicated that 2.5 wt.% of CNFs is the upper limit that can be added to the RTV formulation.

## 1. Introduction

**Figure 1:**
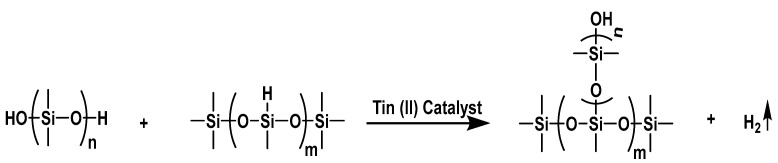
Condensation reaction between Si-OH and Si-H reactive groups promoted by tin (II) catalyst resulting in formation of H_2_ gas and a cross-link.

The condensation reaction between hydroxyl-terminated polydimethylsiloxane (PDMS)(-Si-OH) and a cross-linker containing silane groups (-Si-H) gives rise to hydrogen evolution resulting in a porous cross-linked network. These hydrogen-blown foams are denoted Room-Temperature Vulcanizing (RTV) foams because the condensation reaction, shown in Figure 1, can be readily catalyzed by different catalyst [1,2,3]. Organic tin catalyst; tin (II) 2-ethylhexanoate is the most widely used catalyst for the formation of RTV silicone foams [1,2]. The foams, fabricated using tin (II) catalyst, contain diatomaceous earth to improve mechanical properties, and after curing, are thermally treated to assure completion of chemical reactions. These materials find various uses as gap fillers in ablative heatshields for planetary atmospheric entry [4], sound absorbers [5], piezo-resistive strain sensors [6], and aircraft and automobile engine sealants [7]. However, many studies have shown long-term durability and performance issues due to aging. More specifically, previous work performed by this research team and others has demonstrated that mechanical properties of RTV foams are negatively affected when aged under a compressive load [8,9,10,11].

The tin catalyst promotes the foaming reaction as soon as it is added to the polymer formulation, leaving little time for it to be properly dispersed. One of the ways to overcome this situation is to use a large amount of tin catalyst (5 wt.% of the RTV formulation) to assure that the porous structure is consistently formed. The drawback to this approach is that a high concentration of tin (II) residue is left in the foam, which could contribute to the chemical aging of the material. Previous work has shown that tin (II) 2-ethylhexanoate is susceptible to two chemical aging mechanisms: (1) hydrolysis, where reacting with moisture results in hexanoic acid, and (2) oxidation, where reacting with oxygen changes the oxidation state of tin (II) resulting in tin (IV) species. Specifically, Mössbauer spectroscopy showed an equivalent concentration of tin (II) and tin (IV) residues present in the pristine RTV foam [12], whereas nuclear magnetic resonance (NMR) spectroscopy indicated that the latter are unable to catalyze the condensation reaction between hydroxyl groups [13]. RTV foams are commonly heated to remove low molecular weight residues, but this leaves a high concentration of residual tin (II) and acid in the foam [14]. Consequently, there is a need to identify an effective post-curing procedure that fully oxidizes the active tin (II) species to tin (IV) as well as remove 2-ethylhexanoic acid residues. 

RTV foams often contain 15 wt.% diatomaceous earth (DE), which has been the filler of choice since these foams were first introduced [15,16]. This filler, being a natural and hydrophilic silica, introduces not only chemical composition uncertainty [17], but also moisture residues that could promote hydrolysis of PDMS chains, which negatively impacts lifespan [18]. This has led this research team to investigate hydrophobic fillers, such as Cab-O-Sil TS-720 fumed silica and carbon nanofibers (CNFs), known to offer unique advantages in terms of minimizing environmental interactions [19,20].

In the present work, several new RTV formulations containing DE, TS-720, and CNFs were developed and fully characterized. To effectively fabricate these foams, a post-curing procedure had to first be developed that ensure the deactivation of neat tin (II) 2-ethylhexanoate. Thermogravimetric analysis (TGA) was used to guide the thermal procedure, and ^1^H NMR spectroscopy was used to identify and quantify unreacted material extracted from the post-cured RTV foams. For the filled RTV foams, rheological properties were evaluated to assess changes in flow behavior and processability. Thermomechanical properties, such as maximum tensile strength, resistance to tearing, uniaxial compressibility, specific heat, and thermal conductivity were determined in terms of filler type and concentration. Differential scanning calorimetry (DSC) was used to determine changes to glass transition temperature and crystallization/melting events, and scanning electron microscopy (SEM) was used to characterize the porous structure. Finally, foams were thermally aged under compressive load for three months to determine effectiveness of the proposed post-cure procedure on compression set. This work establishes how different fillers and different filler concentrations affect the thermomechanical properties and aging performance of hydrogen-blown foams, which can be utilized in a variety of multifunctional applications. 

## 2. Materials and Methods 

### 2.1. Materials and Manufacturing

RTV foams were fabricated by combining low, middle, and high molecular weight silanol-terminated polydimethylsiloxanes PLY-7609, 7608, and 7601 (NuSil Technology, LLC, Carpinteria, CA, USA) at concentrations of 49.30, 16.35, and 19.53 wt.%, respectively, with tetrapropylorthosilicate, polymethylhydrogensilane, and diphenylmethylsilanol (2.27, 3.40, and 5.68 wt.%, respectively; NuSil Technology, LLC, Carpinteria, CA, USA). This resin was mixed in a 100 mL polypropylene cup under vacuum at 2000 rpm in an ARV-310 Planetary Mixer (THINKY USA Inc., Laguna Hills, CA, USA). Next, Celite 350 Diatomaceous Earth (DE, Imerys, Paris, France), PDMS-treated fumed silica (TS-720, Cab-O-Sil, Cabot Corporation, Boston, MA, USA), or carbon nanofibers (CNFs, Pyrograff PR-19-XT-PS, Applied Sciences, Cedarville, OH, USA) were added at varying concentrations (0.5 wt.% to 15 wt.%) to the resin, and 2 min mixing at 2000 rpm under vacuum was performed. The resin was then transferred to a 150 mL screw-top mixing container (THINKY USA Inc., Laguna Hills, CA, USA) and stored at 4 °C for 24 h to allow the fillers to fully integrate into the resin. Finally, the resin was mixed for 2 min under vacuum to ensure no settling had occurred, and the desired amount to be catalyzed was transferred to a 100 mL polypropylene cup. 

Tin (II) 2-ethylhexanoate (95%; Thermo Scientific, Waltham, MA, USA) was added to the transferred resin (5 wt.% for all samples), and the mixtures were spun once more at 2000 rpm for 1 min under ambient conditions. The final resin was poured into a flat aluminum mold and cured. Varying curing conditions were explored to determine whether the tin catalyst degraded completely. All curing procedures started at room temperature and utilized 10 °C/min ramp rates. The first procedure mimicked the common post-curing procedure of an isothermal hold at 115 °C for 3.5 h [14]. The second procedure held at 110 °C for 2 h followed by 150 °C for 2 h. The third procedure was identical to the second procedure but also added a final 30 min hold at 200 °C. TGA was performed using a TA Discovery Series TGA 550 (TA Instruments, New Castle, DE, USA), where ~15 mg tin (II) 2-ethylhexanoate droplets were heated according to each of the three curing procedures. All tested samples had ultra-high-purity nitrogen gas flowed across each sample at 40 mL/min, and changes to the masses of each sample were tracked by Trios Software (TA Instruments, New Castle, DE, USA). Once an optimal thermal post-curing profile was determined for the tin catalyst, it was utilized for the fabrication of all RTV foams with and without fillers.

In order to determine the porosities of the RTV foams, it was necessary to obtain their bulk densities. However, it is not possible to obtain a full density foam using the RTV formulation because of porosity resulting from H_2_ generation. Since Sylgard 186 is also a siloxane-based material, it was substituted as the polymer matrix to which DE, TS-720, and CNFs were introduced. Samples for bulk density evaluation were manufactured using Sylgard 186 kits (Dow, Inc., Midland, MI, USA) in a 10:1 ratio (base component/curing component). Varying amounts of the DE, TS-720, and CNF fillers were added corresponding to the filler concentrations in each RTV foam, and samples were cured at room temperature for 5 days. Thus, the bulk density reference for each foam had the same filler concentration as that of the prepared Sylgard 186 samples. The densities of the filled Sylgard 186 samples were determined using an electronic densimeter, model MD-300S (Qualitest, Plantation, FL, USA). The densities of the RTV foams were determined by measuring the volume and mass of the samples. The porosity of the RTV foams was calculated according to Equation (1):

(1) $$porosity=\left(1-\frac{bulk\;density}{density\;of\;porous\;sample}\right)\times 100$$

### 2.2. Material Characterization 

Chemical characterization was performed using proton nuclear magnetic resonance (^1^H-NMR). ^1^H-NMR spectra were collected using a Bruker 400 MHz Avance NMR spectrometer (Bruker, Berlin, Germany) operating at room temperature using a standard Bruker pulse sequence. Chemical shifts were referenced to the CDCl_3_ residue peak at 7.26 ppm. An amount of 200 mg of each foam was immersed in 5 mL of CDCl_3_ (Sigma Aldrich, St. Louis, MO, USA) containing standard dimethyl formamide (DMF) (Alfa Aesar, Ward Hill, MA, USA) for 24 h, and the extracted material was analyzed by ^1^H-NMR spectroscopy. 

Rheological properties of non-catalyzed formulations were determined using a TA Discovery Series Hybrid Rheometer DHR-3 (TA Instruments, New Castle, DE, USA). All experiments were conducted using a 25 mm cross-hatched parallel plate fixture geometry with a working gap of 1000 μm. Stress sweeps were conducted from 10 to 10,000 Pa at an angular frequency of 10 rad/s.

Mechanical properties of the RTV foams were characterized by performing uniaxial compression, tensile, and tear testing on three samples for each test condition using an Instron 3343 Low-Force Testing System (Instron, Norwood, MA, USA) with BlueHill Universal software and a 1 kN load cell. During uniaxial compression testing, each sample was subjected to 4 cycles of compression to a maximum stress of 0.6 MPa at a rate of 0.05 mm/s. The stress–strain curve for each sample was determined by the final cycle, and the displacement reported was normalized to when the instrument first detected an applied load. The stress–strain graph is reported in Supplementary Materials. For tensile testing, each sample was cut using an ASTM D638-V die. All samples were tested at a rate of 1 mm/s. Lastly, tear tests were completed using pneumatic grips with 6 mm upper and lower clevis fittings. Samples were tested at a rate of 500 mm/min following a Type C tear strength test utilizing a Die C punch, as instructed by ASTM D624-00. The tear strength was calculated as the ratio of the applied force to the sample thickness. Additionally, the mechanical response of the foam specimens subjected to cyclic compressive loading was simulated to evaluate the effect of filler type on Mullins softening, and these results are provided in Supplementary Materials.

Thermal characterization was performed on the RTV foams through TGA, DSC, and thermal conductivity measurements. TGA was used to determine the thermal degradation events of the post-cured RTV foams by heating 15 mg samples from 25 °C to 800 °C at a rate of 10 °C/min using the same instrumentation as previously described. DSC experiments were conducted on approximately 5 mg samples of post-cured RTV foam using a TA Discovery Series DSC 2500 (TA Instruments, New Castle, DE, USA). Samples were heated from −150 °C to 0 °C at a rate of 10 °C/min and cycled three times under a 40 mL/min flow of ultra-high-purity nitrogen. Results from the last cycle are presented in this work. The degree of crystallinity was calculated as the ratio of enthalpy of fusion at the melting point to the enthalpy of fusion for a fully crystalline material, which was approximated as 37.4 J/g [20]. Specific heat experiments were conducted on samples of the same approximate weight, cycling from −150 °C to 200 °C at 20 °C/min, holding an isothermal temperature for 10 min at the beginning and end of each run. Three runs were used to demonstrate a range of specific heat values at 25 °C for each foam. Thermal conductivity values of post-cured foams were determined using a TA Fox 50 Heat Flow Meter (TA Instruments, New Castle, DE, USA). In the experiment, the sample was pneumatically compressed (0.41 MPa) between two thermally responsive plates, which equilibrated to 0 °C, 25 °C, 60 °C, 100 °C, 140 °C, and 185 °C, respectively. 

A confocal microscope (Keyence VHX-6000; Keyence Corporation, Osaka, Japan) was used to obtain the thickness of each sample using the Keyence analysis software. A Thermo Fisher Scientific™ Inspect™ 50 (Thermo Fisher Scientific, Waltham, MA, USA) SEM was used to examine the porous structure after 5 nm of gold was sputtered onto the RTV samples using an EMS150R ES Rotary Pumped Coater (Electron Microscopy Sciences, Hatfield, PA, USA). Varying magnification zooms were used (100×–200×) with image voltage operating at 1 kV. SEM images at 100× magnification were used to determine pore size distributions of each foam sample. This was achieved by measuring a sample size of up to 40 pores and plotting the relative frequency of each pore size. Particle size analysis (PSA) of DE and TS-720 was completed by laser diffraction using an Anton Paar PSA 1090 Model D (Anton Paar, AUT, Graz, Austria) instrument. Values for D10, D50, D90, and average particle size were reported. 

### 2.3. Accelerated Aging Study 

RTV foams were compressed using flat fixtures that were placed into an oven at 90 °C and aged for 3 months. The aging fixture consisted of top and bottom aluminum plates that were set to have a constant gap of 0.635 mm, and the foams were compressed by 25%. The thicknesses of the unaged and aged samples were measured to evaluate compression set, which was determined according to:

(2) $$compression\;set=\left(\frac{initial\;thickness-final\;thickness}{initial\;thickness-0.635}\right)\times 100.$$

## 3. Results and Discussion

### 3.1. Thermal Degradation of Tin Catalyst 

**Figure 2:**
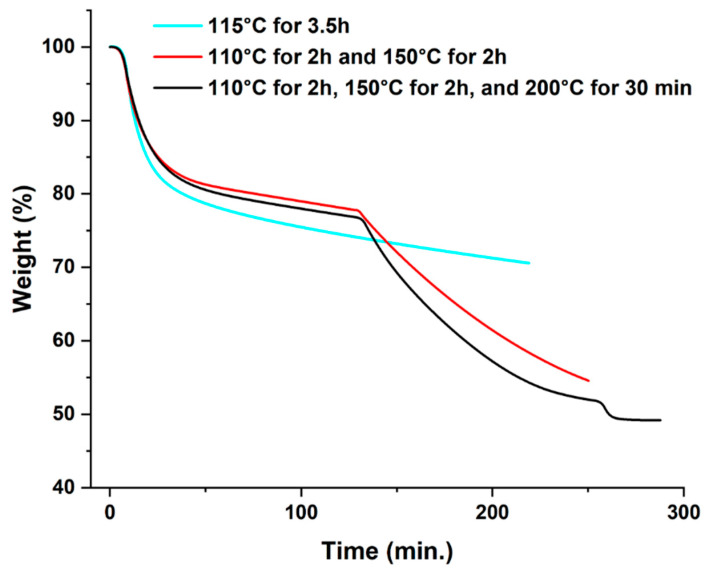
TGA results obtained for the three post-curing conditions for the neat tin catalyst.

The thermal decomposition of neat tin (II) 2-ethylhexanoate was investigated by isothermal TGA experiments where the mass loss of the catalyst was measured as a function of the thermal treatment. Figure 2 shows mass loss for three distinct heating conditions, including the one representing the commonly employed post-curing method (3.5 h at 115 °C) [12]. These results show the ineffectiveness of this approach, since it retains 70.6% residual mass. On the other hand, heating the tin catalyst for 2 h at 110 °C, followed by another 2 h at 150 °C, decreased residual mass to 54.6%. The third treatment consisted in heating the sample for 2 h at 110 °C, 2 h at 150 °C, and 30 min at 200 °C, which resulted in a residual mass of 49.2%. This residual mass corresponds to the SnO_2_ mass percentage in tin (II) 2-ethylhexanoate (the catalyst contains close to 10 wt.% hexanoic acid). While both modified thermal treatments were shown to be more useful than the current one, the most effective treatment was the three-step heating procedure. 

**Figure 3:**
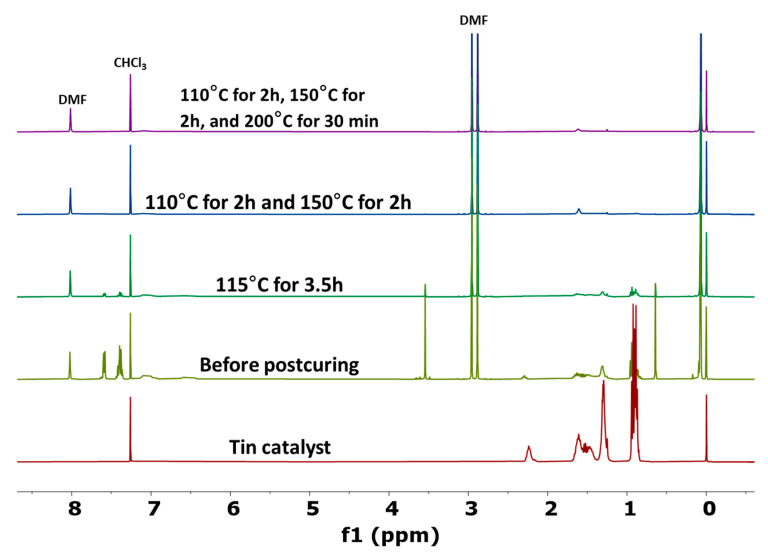
^1^H-NMR spectra of extracts obtained from unfilled RTV foam post-cured under various thermal conditions.

Although TGA results indicated that a modified post-curing procedure is effective in degrading the neat tin catalyst, it is important to evaluate its effectiveness when applied to cured RTV foams. Thus, identical unfilled RTV foams were manufactured and post-cured under the same thermal conditions and extracted in deuterated chloroform (CDCl_3_). The extracted material was analyzed by ^1^H-NMR spectroscopy. Note that only active tin (II) catalyst is soluble in chloroform and hence extractable. Once it is degraded or oxidized to tin (IV) form, it is no longer soluble in chloroform. The ^1^H-NMR spectra of the extracts are shown in Figure 3, where the DMF peak was used as an internal reference. The ^1^H-NMR spectrum of extracted material obtained from the foam before it was post-cured shows peaks associated with unreacted diphenylmethylsilanol (7.3 ppm to 7.7 ppm), low molecular weight silicone prepolymers (0.1 ppm), and active tin catalyst/hexanoic acid (0.8 ppm to 2.2 ppm). The intensity of these peaks decreases with post-curing treatment due to further chemical cross-linking, which means less unreacted material is available to be extracted. The standard post-curing condition of 115 °C for 3.5 h was able to reduce the intensity of these peaks compared to the non-post-cured foam, but the peaks are still noticeable in the NMR spectrum. On the other hand, these peaks are barely discernible when the three-step post-curing procedure is adopted, indicating efficient cross-linking reaction and degradation of active tin (II) catalyst. As a consequence of these results, the three-step post-curing procedure was adopted for the remainder of this work.

### 3.2. RTV Foams Containing 2.5 wt.% Fillers

**Figure 4:**
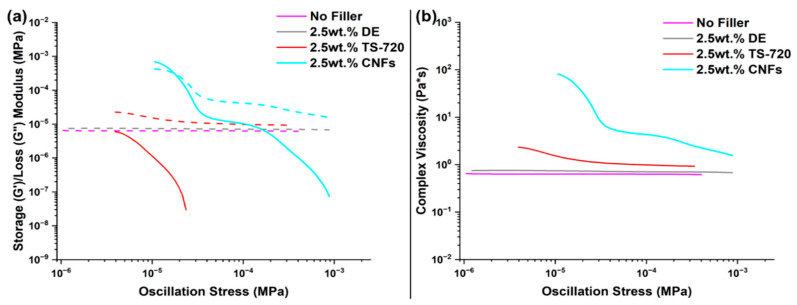
Rheological properties of formulations containing either no filler or 2.5 wt.% filler: (**a**) storage (solid line) and loss moduli (dashed line), and (**b**) η*.

Useful information on flow characteristics are commonly obtained by performing rheological measurements. These experiments are employed to predict practical uses of polymeric formulations by assessing the storage modulus (G′), the loss modulus (G′′), and the complex viscosity (η*, the ratio of stress response to oscillating shear stress). G′ is related to the material’s ability to store energy elastically, whereas G′′ represents the viscous component. When G′′ is greater than G′, the material is considered to be predominantly viscous (it will have a liquid-like character and dissipate more energy than it can store). On the other hand, the polymeric resin will hold its shape while still being able to be extruded when G′ is greater than G′′ [21]. Figure 4 shows G′, G′′, and η* obtained for samples that either have no filler or contain 2.5 wt.% DE, 2.5 wt.% TS-720, or 2.5 wt.% CNFs. The samples containing no filler and 2.5 wt.% DE exhibit similar low G′′, low η*, and undetectable G′, which indicates that these formulations have high flowability. On the other hand, G′ is detected for 2.5 wt.% TS-720 and 2.5 wt.% CNFs formulations, indicative of viscoelastic behavior. Higher G′, G′′, and η* were expected for formulation with 2.5 wt.% DE as hydrophilic filler is expected to have higher interaction with PDMS leading to higher G′, G′′, and η* [21]. To understand this result, the particle size distribution of neat DE was measured, and the average particle size was determined to be 7.46 µm (Table S1 in Supplementary Materials). Thus, we infer that DE particles are too small to significantly increase the viscosity of the resin at 2.5 wt.% concentration. The average particle size of TS-720 is 151.79 µm (Table S1 in Supplementary Materials), helping to explain the observed increase in η*. Specifically, the higher surface area of the suspended particles increases frictional interactions leading to increased η*, which largely guides the foaming behavior. The 2.5 wt.% CNFs formulation has G′ greater than G′′ at low oscillation stresses, which can be attributed to a good interfacial adhesion between the polymer matrix and CNFs (which are a one-dimensional nanofiller comprising a series of graphite planes stacked in the longitudinal direction of the fiber) [22]. These rheological properties indicate that the three filled RTV formulations will be able to be dispensed into a mold without experiencing flowing difficulties, thus assuring their applicability in terms of being useful to fabricate foams of various forms and shapes.

**Figure 5:**
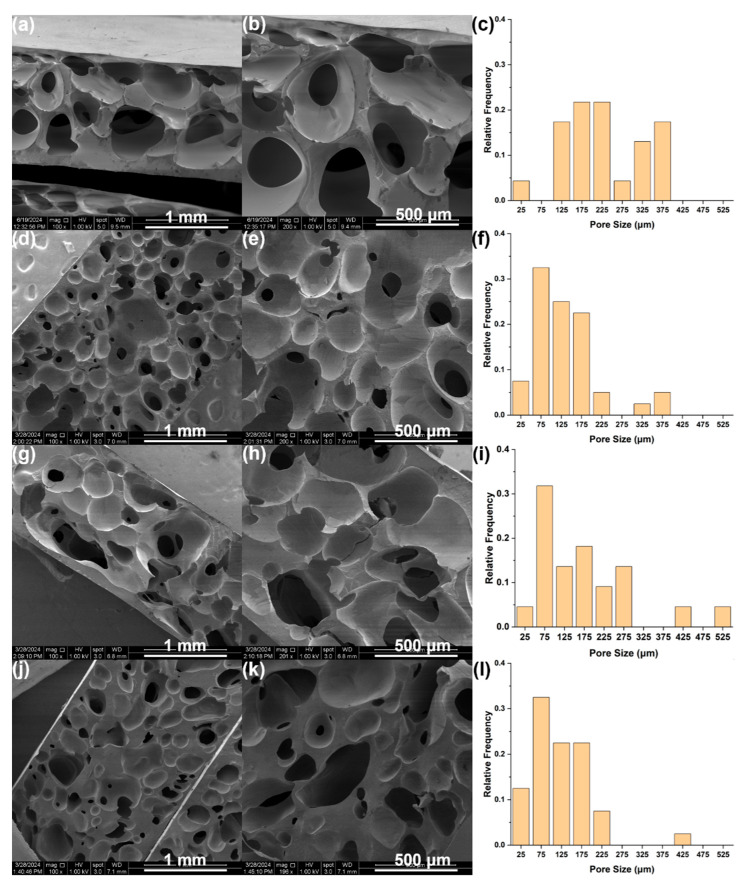
SEM images at 100× and 200×, and corresponding pore distribution, respectively, for RTV foams with (**a**–**c**) no filler, (**d**–**f**) 2.5 wt.% DE, (**g**–**i**) 2.5 wt.% TS-720, and (**j**–**l**) 2.5 wt.% CNFs.

Thermomechanical properties were investigated and are listed in Table 1 and Table 2. Since these properties may be dependent on the porous structure of the foam, the average porosity was determined according to the method described in Section 2.1, whereas pore size distributions were evaluated from SEM images (Figure 5). The average porosity was found to be about 63% for DE foam, 55% for TS-720 foam, 54% for CNF foam, and 67% for the unfilled foam. Pore size distributions and average pore sizes were determined to be similar between foams containing CNFs (122 µm) and DE (136 µm) and slightly larger for foams containing TS-720 (174 µm). The foam with no filler has the largest average pore size (225 µm) and pore size distribution as shown in Figure 5. A more open porous structure could be the result of this formulation having more polymer compared to a formulation containing fillers, or in other words, more hydrogen generation could result in higher porosity.

Mechanical properties, i.e., maximum tensile stress, compressive strain, and tear strength, are listed in Table 1. All fillers yield higher maximum tensile stress and tear strength compared to unfilled samples, with DE producing the smallest increase, followed by TS-720 and then CNFs (see Figure S1a in Supplementary Materials for corresponding plots). This indicates that even at relatively small concentrations, the fillers are impacting the mechanical performance by stiffening the material. Consequently, a stiffer material will also strain less, as shown by the lower compressive strains in the filled materials. It is clear that 2.5 wt.% CNF has a higher reinforcing effect than those of TS-720 and DE, which indicates better polymer–filler interaction. These results point to CNF foams being less prone to physical damage, such as routine handling, than the other three foams. The unfilled foam has the highest porosity and consequently exhibits the highest compressive strain. Table 2 lists a series of thermal properties with corresponding plots in Figure S1 in Supplementary Materials. CNF foam exhibits the highest thermal conductivity at any temperature, which is expected because CNFs have higher thermal conductivity (ranges between 28 and 43 W/mK) [23] than DE or TS-720 (below 0.02 W/mK) [24]. On the other hand, the influence of CNFs on specific heat was indistinct due to the large range of values measured. This is likely due to small sample sizes used in this experiment (about 5 mg), which demonstrates sample variability at this scale. Thermal degradation was measured by TGA experiments, where the onset of degradation is defined by Td^5%^. This value is higher for TS-720 (373 °C) and CNF foams (375 °C) than for foams with no filler (360 °C) or with DE (362 °C). The first derivative (DTG) of the TGA curve determines the maximum peak temperature of mass-loss, which was lowest for the unfilled foam. These results demonstrate that the overall thermal stability improves when inorganic fillers are introduced into the polymer matrix. Finally, DSC experiments provided information on the effect of filler types on glass transition temperature (T_g_), crystallization, and melting events. T_g_, which depends on the degree of freedom of the segmental motion, cross-linking, and entanglement constrains, is slightly higher for the 2.5 wt.% CNFs foam. Also, it is interesting to notice that the addition of DE to the polymer network decreased crystallization, with TS-720 completely quenching the crystallization of the PDMS chains. The reason for decreasing crystallinity is likely due to fillers disturbing the ability of polymer chains to align to form crystalline domains [25]. Interestingly, the opposite effect occurred for the 2.5 wt.% CNFs foam, where CNFs acted as a heterogeneous nucleation agent, thus increasing crystallization of PDMS chains [26]. 

### 3.3. RTV Foams Containing up to 3.5 wt.% CNFs

**Figure 6:**
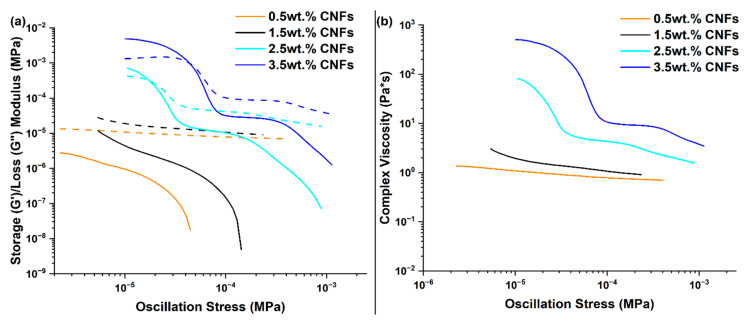
Rheological properties of RTV foams containing various CNF concentrations: (**a**) G′ (solid line) and G′′ (dashed line) and (**b**) η*.

Since the formulation containing 2.5 wt.% CNFs exhibited a viscoelastic component and the resulting foam exhibited increased tensile and tear strength compared to other fillers, additional formulations containing 0.5 wt.%, 1.5 wt.%, and 3.5 wt.% CNFs were prepared to further investigate these behaviors. Rheological properties of these formulations are shown in Figure 6, where increasing values of G′, G′′, and η* are observed with increasing CNF concentration. The sample containing 3.5 wt.% CNFs exhibited the highest rheological values, and it presented flow difficulties when used to mold the foam, resulting in a non-uniform foam. As a result, this formulation was not investigated further in this work, and it provides upper limit values for G′, G′′, and η* regarding the practical use of novel RTV formulations.

**Figure 7:**
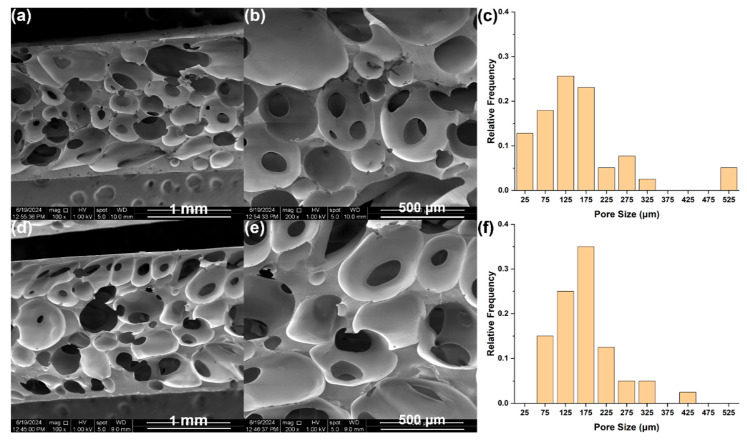
SEM Images at 100× and 200×, and corresponding pore distribution, respectively, for RTV foams with (**a**–**c**) 0.5 wt.% CNFs, and (**d**–**f**) 1.5 wt.% CNFs.

The average porosity was determined as about 67% for foams containing 0.5 wt.% and 1.5 wt.% CNFs. Pore size distributions and average pore sizes were determined by SEM (Figure 7). Pore size distributions were similar for both foams, with average pore sizes being 158 µm for 0.5 wt.% CNFs and 171 µm for 1.5 wt.% CNFs foams. 

Results for maximum tensile stress, compressive strain, and tear strength are listed in Table 3 (see Figure S2a in Supplementary Materials for corresponding plots). As CNF concentration in the polymer matrix increases, maximum tensile stress and tear strength increase, indicative of the mechanical reinforcing effect of CNFs. This stiffening behavior from the added filler is also seen in the compressive tests, where the compressive strain at 0.6 MPa decreases with higher filler concentrations. Table 4 lists thermal properties with corresponding plots in Figure S2 in Supplementary Materials. Increasing CNF concentration from 0.5 wt.% to 2.5 wt.% has a negligible effect on T_g_ and crystallization/melting events, thermal conductivity, and specific heat. 

### 3.4. RTV Foams Containing a Combination of DE and CNFs

**Figure 8:**
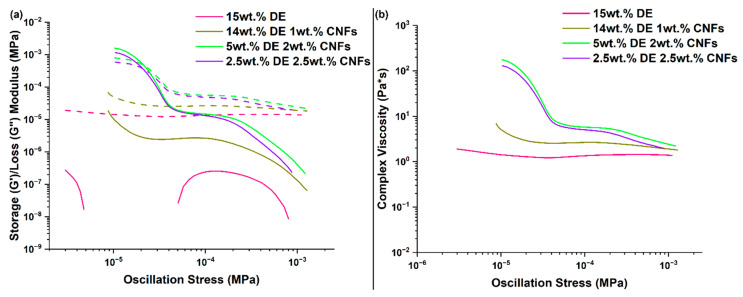
Rheological properties of RTV foams containing a combination of DE and CNFs: (**a**) G′ (solid line) and G′′ (dashed line), and (**b**) η*.

Formulations combining DE and CNFs into the polymer matrix were investigated to further understand the role of diverse inorganic fillers on rheological properties. The maximum concentration of DE was kept at 15 wt.%, which corresponds to the concentration routinely used to formulate these RTV foams. The maximum CNF concentration was kept at 2.5 wt.% since the resin would not flow appropriately at higher concentrations. Thus, three formulations containing 14 wt.% DE and 1 wt.% CNFs, 5 wt.% DE and 2 wt.% CNFs, and 2.5 wt.% DE and 2.5 wt.% CNFs were prepared and compared to a formulation containing 15 wt.% DE. Figure 8 shows G′, G′′, and η* as a function of oscillation stress for these formulations. Unlike what was previously observed for 2.5 wt.% of DE formulations, G′ started to appear in 15 wt.% though not fully. This suggests that increasing DE concentration from 2.5 wt.% to 15 wt.% promotes the onset of viscoelastic behavior. This behavior becomes much more pronounced with the addition of CNFs to formulations containing DE due to reinforcing effect of CNFs. In particular, rheological properties of formulations containing 5 wt.% DE and 2 wt.% CNFs and 2.5 wt.% DE and 2.5 wt.% CNFs have nearly identical trends across the stress sweep. Both formulations flowed easily into the mold and were able to be adequately spread throughout. The 14 wt.% DE and 1 wt.% CNFs formulation exhibited G′, G′′, and η* values between the other three formulations and flowed extremely well into the mold. These results demonstrate that, even at low CNF concentrations, this filler has a greater reinforcing effect than DE due to better polymer–filler interaction and aspect ratio [27].

**Figure 9:**
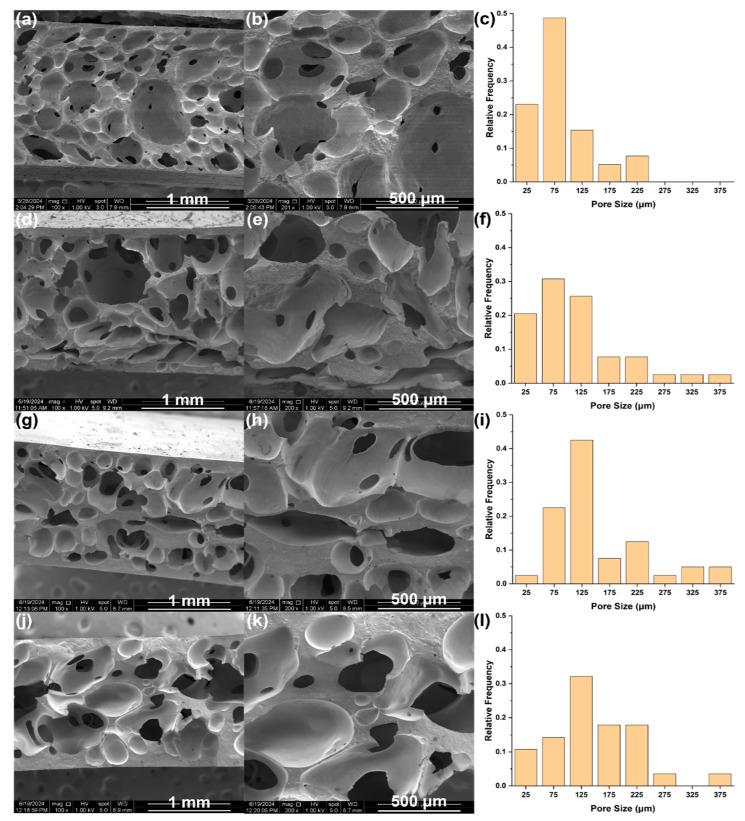
SEM Images at 100× and 200×, and corresponding pore distribution, respectively, for RTV foams with (**a**–**c**) 15 wt.% DE, (**d**–**f**) 14 wt.% DE+ and 1 wt.% CNFs, (**g**–**i**) 5 wt.% DE and 2 wt.% CNFs, (**j**–**l**) 2.5 wt.% DE and 2.5 wt.% CNFs.

The average pore size distributions were obtained by SEM and are shown in Figure 9. The average pore sizes estimated from these images are 86 µm for 15 wt.% DE foam, 117 µm for 14 wt.% DE and 1 wt.% CNFs, 153 µm for 5 wt.% DE and 2 wt.% CNFs, and 149 µm for 2.5 wt.% DE and 2.5 wt.% CNFs. These results show that high DE concentrations generate smaller pore sizes and pore size distributions.

Results for maximum tensile stress, compressive strain, and tear strength are listed in Table 5 (see Figure S3a in Supplementary Materials for corresponding plots). The foam containing 14 wt.% DE and 1 wt.% CNFs outperforms the other three foams in terms of maximum tensile stress (0.345 MPa) and tear strength (103 N/m). When compared with the 15 wt.% of DE, it can be noted that replacing 1 wt.% of DE with CNF (i.e., 14 wt.% DE and 1 wt.% CNFs) led to significant improvement in the mechanical properties of the resulting foam. This could be attributed to the higher reinforcing effect of the CNFs compared to DE. However, the reinforcing effect of the CNFs could be shadowed if the overall filler content is very high regardless of the amount of CNFs. For example, the maximum tensile strength and tear strength for 15 wt.% of DE-containing foam is higher than that of foams with 5 wt.% DE + 2 wt.% CNFs and 2.5 wt.% DE + 2.5 wt.% CNFs which have overall filler content of 7 wt.% and 5 wt.%, respectively. Table 6 lists thermal properties with corresponding plots in Figure S3 in Supplementary Materials. It is clear that the addition of CNFs has little effect on T_g_ and crystallization, but foams containing 2.5 wt.% CNFs exhibit better thermal stability compared to foams with lower CNF concentration or absence of CNFs. In addition, foams with 2.5 wt.% CNFs have slightly higher thermal conductivity and lower specific heat. From an applied perspective, the potential material property benefits and material inconsistencies offered by using less DE in favor of CNFs must be balanced with cost (while DE costs ~$0.18/g [28], CNFs cost ~$7/g [29]).

### 3.5. Aging Studies

Our previous work demonstrated that reactive residues promote chemical changes in the polymer, which resulted in compression set in RTV silicone foams thermally aged under 25% compressive load [8]. We reported that foams aged for 2000 days at 90 °C exhibit close to 25% compression set. In addition, Patel and co-workers have identified two degradation mechanisms with activation energies of 21.82 ± 6.85 kJ/mol (for temperatures below 150 °C) and 76.90 ± 44.53 kJ/mol (for temperatures above 150 °C) for similar RTV silicone foams aged under thermal treatments [18]. In Patel’s work, time–temperature superposition and Arrhenius treatment generated a master curve showing compression set versus aging time, which, for instance, showed compression set to be about 3% when the foam is aged for 2000 h at 25 °C. Since in the present work foams were thermally aged for 3 months (2160 h) at 90 °C, this condition cannot be directly compared with foams aged at 25 °C. However, knowing the activation energy of the degradation mechanism allows one to estimate the ratio between the rate constant at 25 °C versus the rate constant at 90 °C, which turns out to be about 4.9 (using 22 kJ/mol as the activation energy). Thus, aging at 90 °C for 2160 days corresponds to aging at 25 °C for about 10,600 h. This aging condition would give rise to compression set close to 5%, according to the master curve shown in Patel’s study. The 22 ± 7 kJ/mol activation energy has been associated with acid catalyzed hydrolysis [28,30], which will result in chain scission and recombination of chains in the polymer matrix. This degradation mechanism would give rise to compression set if it takes place when the foam is under a compressive load. In the present work, no compression set was detected for the aged RTV foams that were post-cured following the three-step heating procedure. Thus, we conclude that this post-curing approach was beneficial to mitigate aging by removing acid residues and oxidizing catalytically active tin (II) residues.

## 4. Conclusions

In this work, a series of RTV silicone foams were synthesized to understand the effect of distinct inorganic fillers (DE, TS-720, and CNF) on thermomechanical properties. Prior to the manufacturing of the foams an effective approach to deactivate tin (II) residues originating from the catalyst was developed. TGA and NMR experiments guided the selection of a three-step post-curing approach: 2 h at 110 °C, 2 h at 150 °C, and 30 min at 200 °C. Fabricated RTV foams containing no fillers exhibited large average pore sizes (225 µm), high porosity (67%), low tear strength (23.9 N/m), and low tensile strength (0.039 MPa). On the other hand, foams containing up to 2.5 wt.% CNFs had smaller pore sizes (122 µm), lower porosity (54%), higher tear strength (52.6 N/m), and higher tensile strength (0.15 MPa). The maximum CNF amount that could possibly be added to the RTV silicone formulation was less than 3.5 wt.%; otherwise, the resin did not flow properly, and low-quality foams were produced. RTV silicone foams synthesized with a combination of 14 wt.% DE and 1 wt.% CNFs exhibited the highest mechanical properties in terms of tensile strength (0.345 MPa) and tear strength (103 N/m). 

## Figures and Tables

**Table 1:** Mechanical properties of unfilled foam and foams containing 2.5 wt.% fillers.

RTV Foam with	Maximum Tensile Stress (MPa)	Compressive Strain at 0.6 MPa (%)	Tear Strength (N/m)
No Filler	0.039 ± 0.010	81.1 ± 5.5	23.9 ± 0.1
2.5 wt.% DE	0.057 ± 0.001	70.5 ± 6.5	27.5 ± 5.8
2.5 wt.% TS-720	0.089 ± 0.010	61.0 ± 1.2	35.8 ± 1.8
2.5 wt.% CNFs	0.150 ± 0.020	67.9 ± 2.6	52.6 ± 1.8

**Table 2:** Thermal properties of unfilled foam and foams containing 2.5 wt.% fillers.

RTV Foam with	Thermal Conductivity at 25 °C (W m^−1^ K^−1^)	Td^5%^ (°C)	Derivative Peak (°C)	Glass Transition (°C)	Melting Peak (°C)	Crystallization Peak (°C), % Crystallization (X_c_)	Specific Heat at 25 °C (J g^−1^ C^−1^)
No filler	0.10	360	401	−124	−53	−96, 19.2	1.48–1.65
2.5 wt.% DE	0.11	362	420	−124	−54	−97, 7.3	1.45–1.53
2.5 wt.% TS-720	0.10	373	416	−123	N/A	N/A, N/A	1.22–1.50
2.5 wt.% CNFs	0.12	375	417	−118	−51	−87, 25.8	1.43–1.54

**Table 3:** Mechanical properties of RTV foams containing up to 2.5 wt.% CNFs.

RTV Foam with	Maximum Tensile Stress (MPa)	Compressive Strain at 0.6 MPa (%)	Tear Strength (N/m)
0.5 wt.% CNFs	0.072 ± 0.009	75.9 ± 2.7	27.9 ± 5.3
1.5 wt.% CNFs	0.089 ± 0.002	74.3 ± 2.7	30.2 ± 2.9
2.5 wt.% CNFs	0.150 ± 0.020	67.9 ± 2.6	52.6 ± 1.8

**Table 4:** Thermal properties of RTV foams containing up to 2.5 wt.% CNFs.

RTV Foam with	Thermal Conductivity at 25 °C (W m^−1^ K^−1^)	Td^5%^ (°C)	Derivative Peak (°C)	Glass Transition (°C)	Melting Peak (°C)	Crystallization Peak (°C), % Crystallization (X_c_)	Specific Heat at 25 °C (J g^−1^ C^−1^)
0.5 wt.% CNFs	0.10	369	410	−120	−51	−89, 26.4	1.42–1.54
1.5 wt.% CNFs	0.10	370	425	−119	−51	−89, 26.3	1.33–1.60
2.5 wt.% CNFs	0.12	375	417	−118	−51	−87, 25.8	1.43–1.54

**Table 5:** Mechanical properties of RTV foams containing a combination of DE and CNFs.

RTV Foam with	Maximum Tensile Stress (MPa)	Compressive Strain at 0.6 MPa (%)	Tear Strength (N/m)
15 wt.% DE	0.215 ± 0.020	63.2 ± 0.8	76.8 ± 5.1
14 wt.% DE + 1 wt.% CNFs	0.345 ± 0.040	68.1 ± 2.6	103.1 ± 5.0
5 wt.% DE +2 wt.% CNFs	0.311 ± 0.030	61.5 ± 4.2	73.0 ± 0.1
2.5 wt.% DE +2.5 wt.% CNFs	0.155 ± 0.010	67.8 ± 2.6	63.9 ± 1.8

**Table 6:** Thermal properties of RTV foams containing a combination of DE and CNFs.

RTV Foam with	Thermal Conductivity at 25 °C (W m^−1^ K^−1^)	Td^5%^ (°C)	Derivative Peak (°C)	Glass Transition (°C)	Melting Peak (°C)	Crystallization Peak (°C), % Crystallization (X_c_)	Specific Heat at 25 °C (J g^−1^ C^−1^)
15 wt.% DE	0.10	359	412	−120	−52	−87, 18.9	1.55–1.58
14 wt.% DE + 1 wt.% CNFs	0.10	363	407	−119	−51	−84, 19.9	1.42–1.59
5 wt.% DE +2 wt.% CNFs	0.11	370	416	−120	−50	−85, 26.5	1.40–1.51
2.5 wt.% DE +2.5 wt.% CNFs	0.11	371	414	−118	−50	−85, 26.4	1.20–1.37

## Data Availability

The authors confirm that the data supporting the findings of this study are available within the article.

## Supplementary Materials

The following supporting information can be downloaded at: https://www.mdpi.com/article/10.3390/polym17141998/s1. References [31,32,33] are cited in the Supplementary Materials.
