# Supplementary material for: Relationship Between Filler Type, Thermomechanical Properties, and Aging of RTV Silicone Foams
Source: Polymers (Basel). 2025 Jul 21;17(14):1998. doi: 10.3390/polym17141998 (PMC12300107; doi:10.3390/polym17141998)
Supplement: Supplementary file 1 [file polymers-17-01998-s001.zip › polymers-3703683-supplementary.pdf]

Relationship between Filler Type, Thermomechanical Properties, and Aging of RTV Silicone Foams

Xavier M. Torres[a], John R. Stockdale[a], Adam Pacheco[a], Shelbie A. Legett[a], Lindsey B. Bezek[a], Bart Benedikt[b], Andrea Labouriau[a], and Santosh Adhikari*[a]

[a]C-CDE: Chemical Diagnostics and Engineering Group, Los Alamos National Laboratory, Los Alamos, NM 87545, USA.

[b]W-13: Advanced Engineering Analysis, Los Alamos National Laboratory, Los Alamos, NM 87545, USA

Table S1. Particle size distribution of diatomaceous earth (DE) and TS-720.

Sample	D10 (µm)	D50 (µm)	D90 (µm)	Mean (µm)
DE	0.10	1.36	23.57	7.46
TS-720	24.24	115.73	323.31	151.79

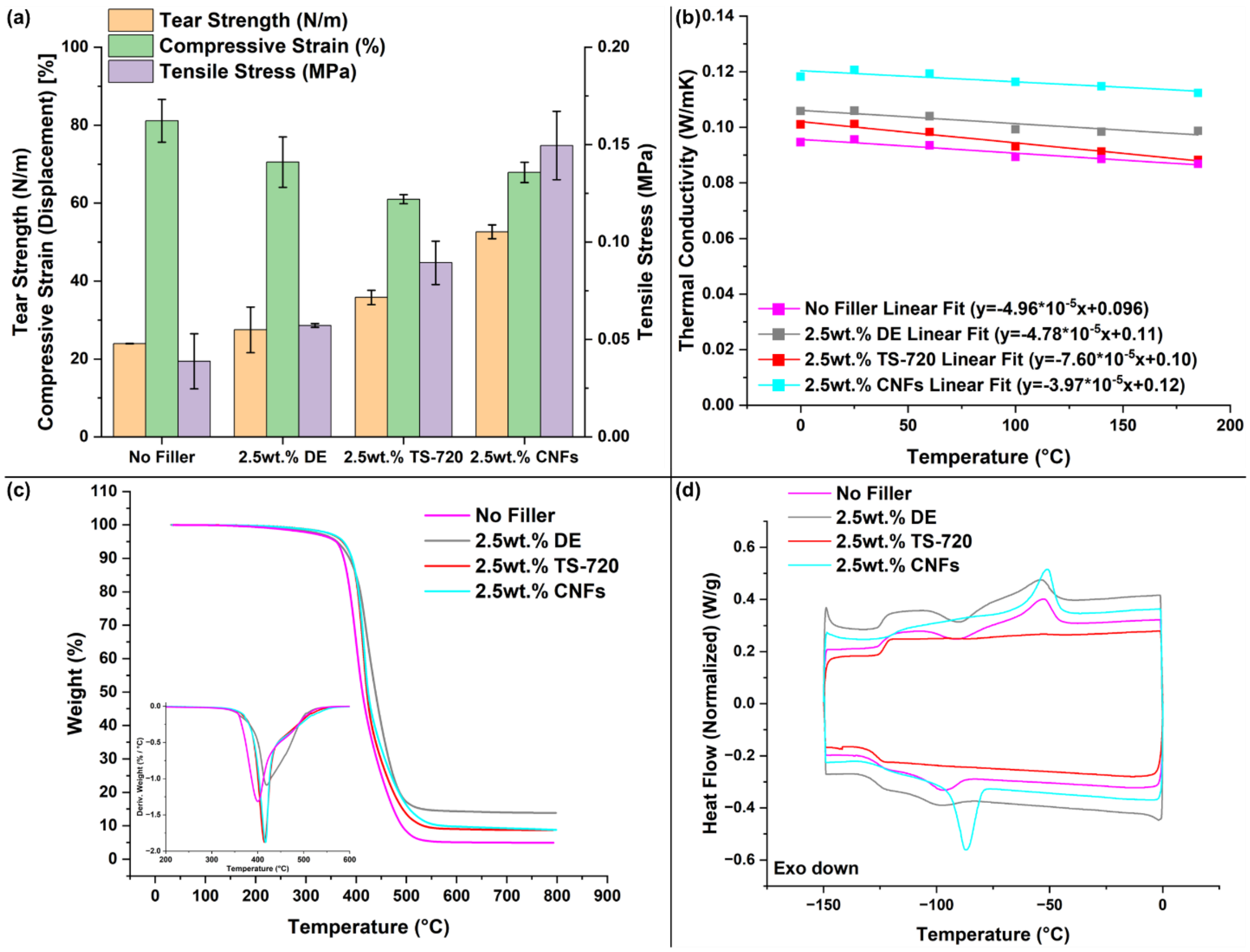

Figure S1. No filler and 2.5wt.% filler samples: (a) tear strength, compressive strain at 0.6 MPa, and maximum tensile stress, (b) thermal conductivity, (c) TGA/dTGA results, and (d) DSC results.

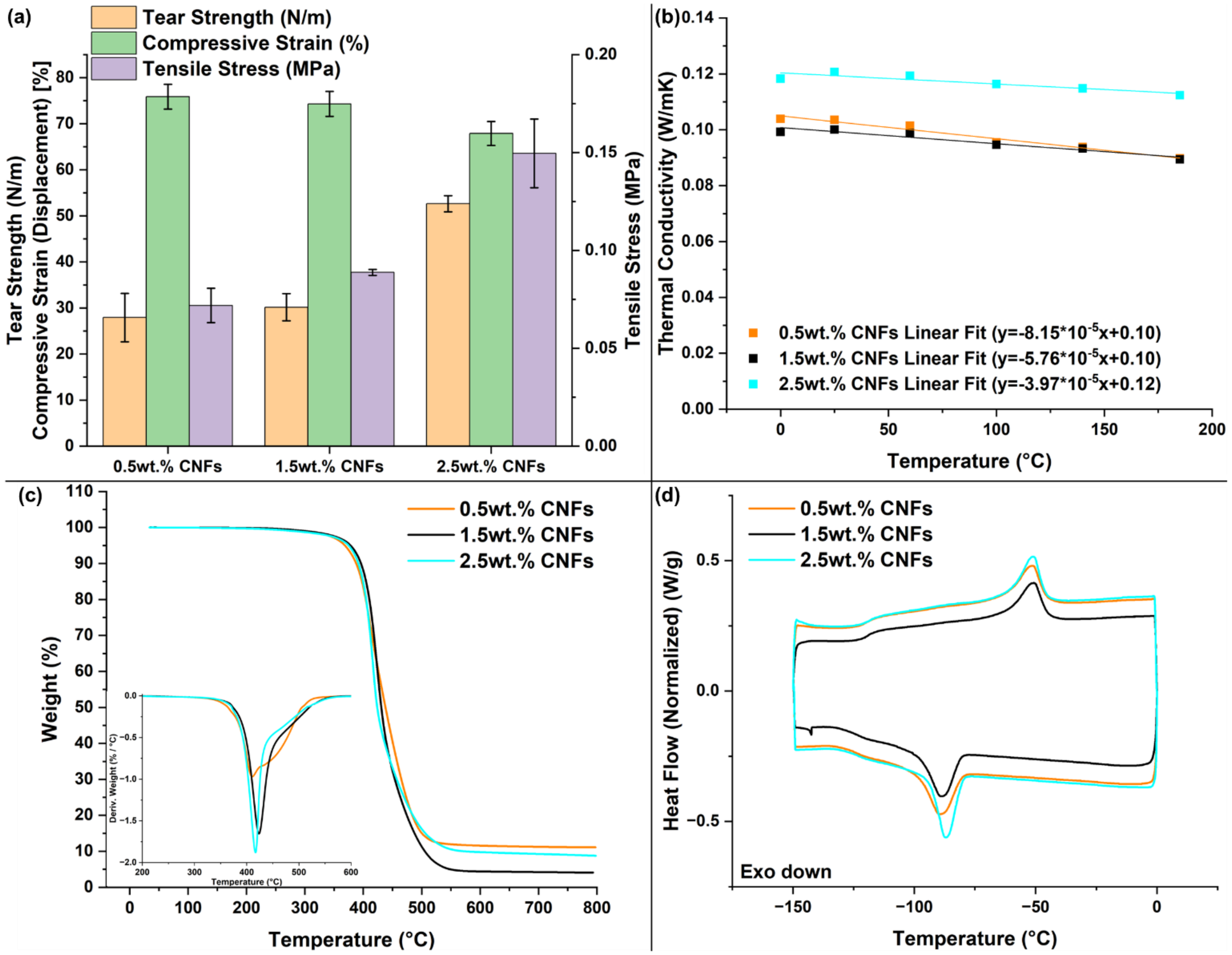

Figure S2. Samples with different filler concentrations of CNFs: (a) tear strength, compressive strain at 0.6 MPa, and maximum tensile stress, (b) thermal conductivity, (c) TGA/dTGA results, and (d) DSC results.

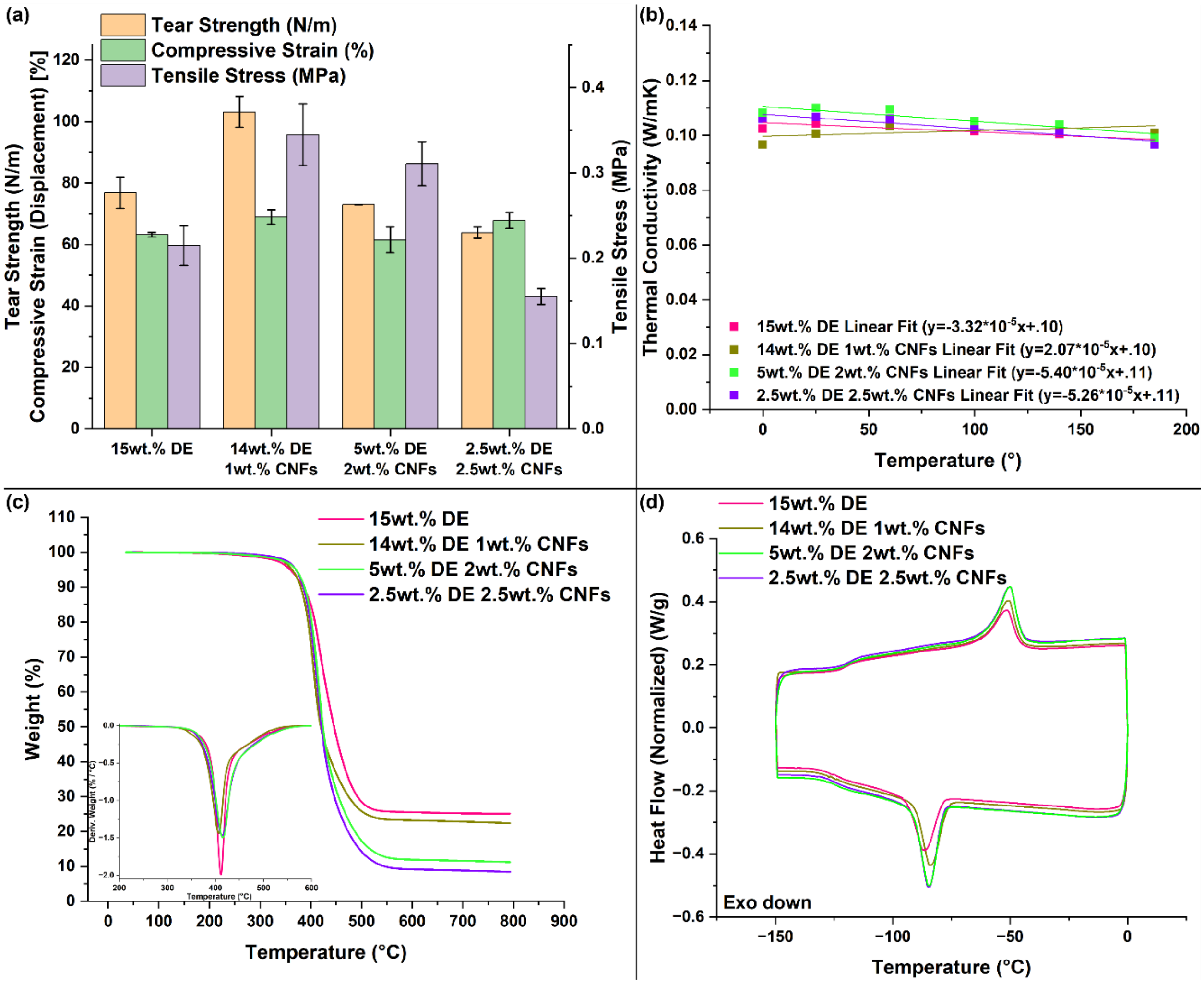

Figure S3. Samples with different filler concentrations of DE and CNFs: (a) tear strength, compressive strain at 0.6 MPa, and maximum tensile stress, (b) thermal conductivity, (c) TGA/dTGA results, and (d) DSC results.

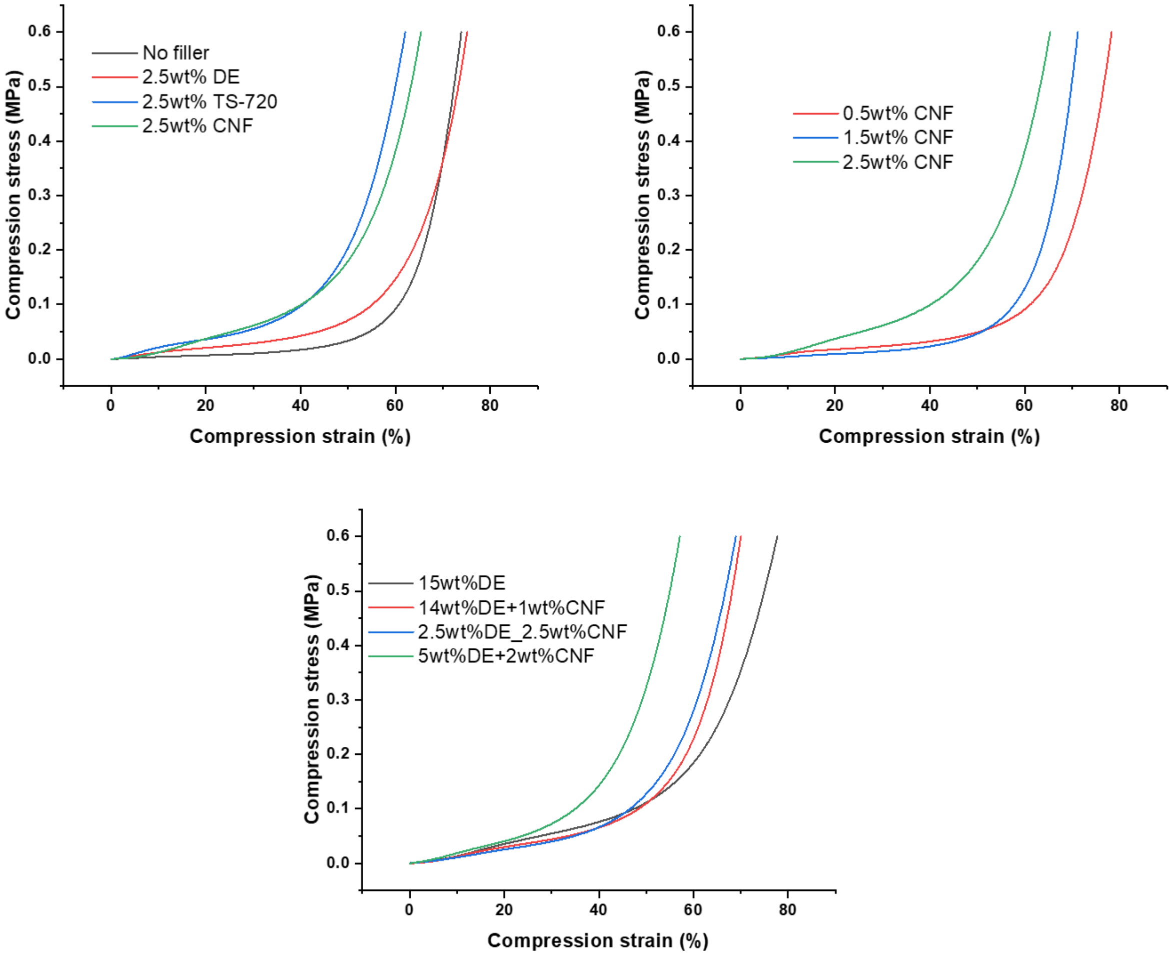

Figure S4. Compression strain-strain curves for samples with different fillers.

Filler Type Effect on Mullins Softening

The phenomenon characterized by a softening of the mechanical properties of a rubber-like material after it is mechanically tested is known as the Mullins effect. It is characterized by a lower resulting stress for the same applied strain and typically appears after the first load (on tensile or compressive tests)[1]. The mechanical response of the foam specimens subjected to the performed cyclic compressive loading was simulated using two models, namely the hyperfoam and Ogden-Roxburgh models. Both models were used as implemented in ABAQUS finite element software. First, the reversible hyperelastic response was modeled using the hyperfoam model[2]. Hyperfoam is a modification of the Ogden hyperelastic model, and it uses the isochoric principal stretch formulation ($\hat{\lambda}_i\ , i = 1, 2, 3$) to define the strain energy density function U. In the hyperfoam formulation, the strain energy density function U is expanded into a series up to a user-specified maximum number of terms N. The coefficients of this expansion are determined by fitting the model to the experimental stress vs. strain curves.

$$U\left(\hat{\lambda}_1, \hat{\lambda}_2, \hat{\lambda}_3, J^{el}\right) = \sum_{i=1}^{N} \frac{2\mu_i}{\alpha_i^2}\left[\hat{\lambda}_1^{\alpha_i} + \hat{\lambda}_2^{\alpha_i} + \hat{\lambda}_3^{\alpha_i} - 3 + \frac{1}{\beta_i}\left((J^{el})^{-\alpha_i\beta_i} - 1\right)\right] \quad (1)$$

J^{el} is the Jacobian (material volume change), and α_i, β_i, and μ_i are hyperfoam material model parameters. In this work, the hyperfoam model was calibrated by fitting the model to the first compressive loading stress vs. strain curve. The first loading curve was used for hyperfoam model calibration because the first loading is not affected by Mullins damage. The numerical values of the fitted material parameters α_i, β_i, and μ_i are provided in Table S2.

Table S2. Hyperfoam calibrated material parameters α_i, β_i, and μ_i obtained by fitting the hyperfoam model to the test data.

RTV Foam with	Hyperfoam Parameters $\boldsymbol{\mu_i}$	Hyperfoam Parameters $\boldsymbol{\alpha_i}$	Hyperfoam Parameters $\boldsymbol{\beta_i}$
No Filler	μ_1= -0.607, μ_2=0.635, μ_3=0.0045, μ_4=0.0004	α_1=11.7, α_2=13.0, α_3=7.4, α_4=-3.1	β_1=0.0, β_2=0.0, β_3=0.0, β_4=0.0
2.5wt.% DE	μ_1=-0.713, μ_2=0.717, μ_3=0.178, μ_4=-0.0014	α_1=1.4, α_2=3.3, α_3=-1.8, α_4=-3.8	β_1=0.0, β_2=0.0, β_3=0.0, β_4=0.0
2.5wt.% TS-720	μ_1=-1.008, μ_2=0.887, μ_3=0.362, μ_4=-0.06	α_1=0.46, α_2=2.5, α_3=-2.2, α_4=-3.7	β_1=0.0, β_2=0.0, β_3=0.0, β_4=0.0
2.5wt.% CNFs	μ_1=4.492, μ_2=-3.475, μ_3=-1.185, μ_4=-0.26	α_1=18.3, α_2=25.0, α_3=8.2, α_4=-0.28	β_1=0.0, β_2=0.0, β_3=0.0, β_4=0.0

The Mullins damage was modeled using the Ogden-Roxburgh model[3]. Detailed discussion of the Ogden-Roxburgh model is beyond the scope of this work; we will only mention that the Ogden-Roxburgh model is an energy dissipation mechanism that is activated when the current strain energy density function U is larger than the maximum energy U^{max} reached during the entire deformation history. The amount of energy dissipation is calculated from the damage function η given by:

$$\eta = 1 - \frac{1}{r}\operatorname{erf}\left(\frac{U^{max} - U}{m + \beta U^{max}}\right), \quad (2)$$

where erf is error function, and r, m and β are material parameters to be calibrated from the experimental data.

In this work, we attempted to calibrate the Ogden-Roxburgh using the first and the second experimental loading stress vs. strain curves only. The model parameters are provided in Table S3. It is noted that parameter m was assumed to be zero for all foams. To calibrate parameter m, multiple experimental loading-unloading curves with varying peak stress are needed. In addition, parameter β was also kept constant at 2.5. This parameter governs the amount of softening around the peak stress (0.6 MPa in the present case) and for the measured data for all studied foams, we did not notice measurable softening at peak stress. Decreasing the r parameter in Table S3 indicates a stronger Mullins effect. If there was no Mullins damage, the first and second loading curves would be identical. The second loading is expected to be softer than the first one due to Mullins energy dissipation. Figure S4 shows the stress-strain response of unfilled and 2.5wt.%filled foams obtained for the first and second uniaxial compression cycles. Weak Mullins damage

is observed for the foam containing no filler, whereas the largest effect is observed for the foam containing 2.5 wt% CNFs.

Table S3. Ogden-Roxburgh model parameters used to model the Mullins effect in the tested foams.

RTV Foam with	Ogden-Roxburgh Parameter *r*	Ogden-Roxburgh Parameter *m*	Ogden-Roxburgh Parameter β
No Filler	No measurable Mullins effect	--	--
2.5wt.% DE	4.0	0.0	2.5
2.5wt.% TS-720	4.5	0.0	2.5
2.5wt.% CNFs	3.1	0.0	2.5

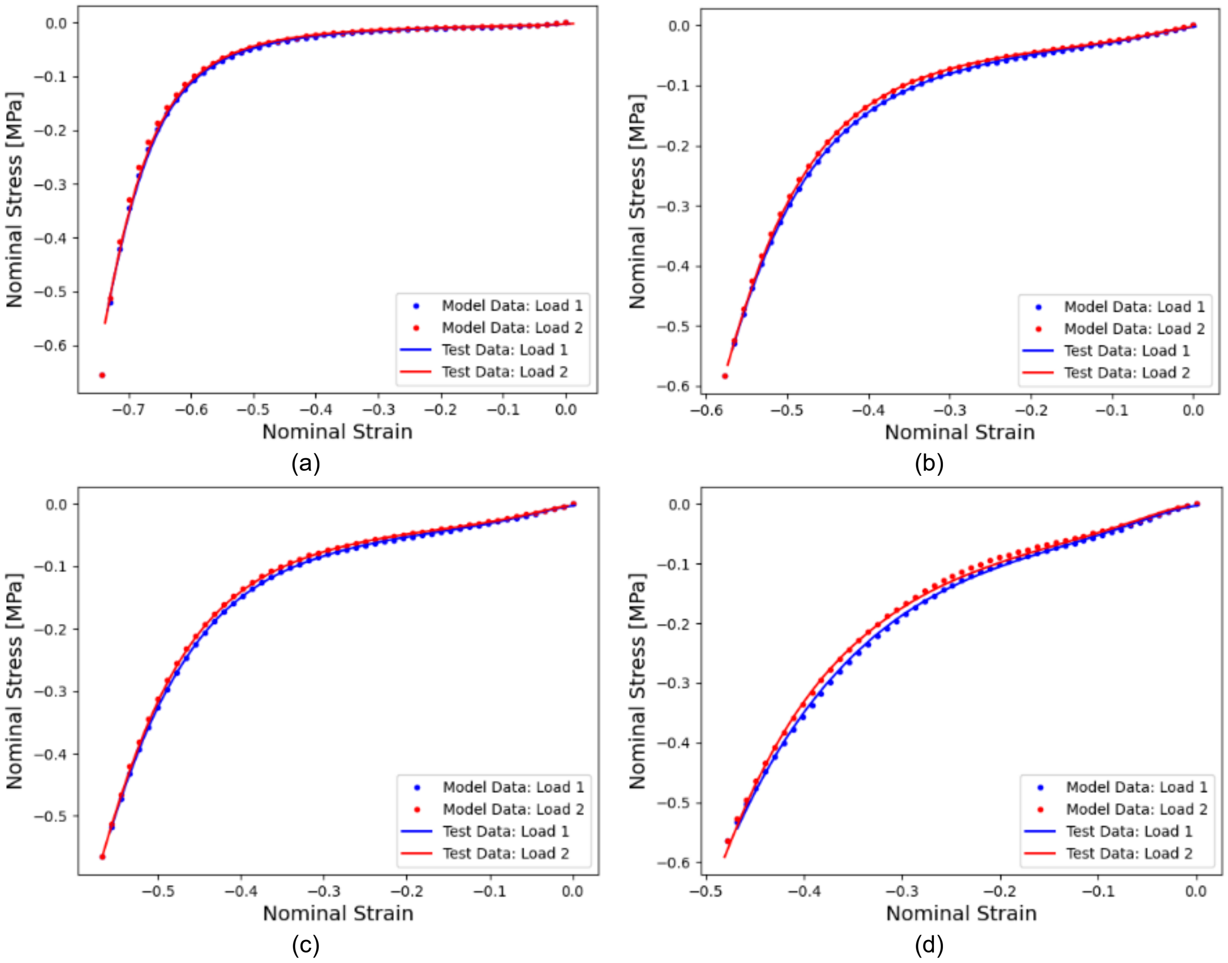

Figure S5. Stress-strain behavior of a) unfilled foam, b) 2.5 wt.% DE foam, c) 2.5 wt.% TS-720 foam), and d) 2.5 wt.% CNFs foam. The first ("blue") and second ("red") compressive loads are shown in each plot.

References

(1) Diani, J.; Fayolle, B.; Gilormini, P. A review on the Mullins effect. *Eur Polym J* **2009**, *45* (3), 601-612. DOI: 10.1016/j.eurpolymj.2008.11.017.

(2) Briody, C.; Duignan, B.; Jerrams, S.; Tiernan, J. The implementation of a visco-hyperelastic numerical material model for simulating the behaviour of polymer foam materials. *Comp Mater Sci* **2012**, *64*, 47-51. DOI: 10.1016/j.commatsci.2012.04.012.

(3) Ogden, R. W.; Roxburgh, D. G. A pseudo-elastic model for the Mullins effect in filled rubber. *P Roy Soc a-Math Phy* **1999**, *455* (1988), 2861-2877. DOI: DOI 10.1098/rspa.1999.0431.
